# Raised circulating tenascin-C in rheumatoid arthritis

**DOI:** 10.1186/ar4105

**Published:** 2012-11-29

**Authors:** Theresa H Page, Peter J Charles, Anna M Piccinini, Vicky Nicolaidou, Peter C Taylor, Kim S Midwood

**Affiliations:** 1 The Kennedy Institute of Rheumatology, Nuffield Department of Orthopaedics, Rheumatology and Musculoskeletal Sciences, Oxford University, 65 Aspenlea Road, London, W6 8LH, UK

## Abstract

**Introduction:**

The aim of this study was to examine whether circulating levels of the pro-inflammatory glycoprotein tenascin-C (TNC) are elevated in musculoskeletal disorders including rheumatoid arthritis (RA) and to assess in RA whether levels are related to clinical disease status and/or patient response to treatment.

**Methods:**

TNC in serum or plasma was quantified by ELISA. Samples from 4 cohorts of RA patients were examined and compared to normal human subjects and to patients with other inflammatory diseases.

**Results:**

Circulating TNC levels were significantly raised in patients with RA, as well as patients with systemic lupus erythematosus, idiopathic inflammatory myositis, psoriatic arthritis and ankylosing spondylitis, whilst patients with Sjogren's syndrome displayed levels similar to healthy controls. The highest levels of TNC were observed in RA patients with late stage disease. In early disease TNC levels correlated positively with ultrasound determined erosion scores. Treatment of early RA patients with infliximab plus methotrexate (MTX) resulted in a transient decrease in circulating TNC over the first year of therapy. In contrast, TNC levels increased over time in RA patients receiving MTX alone. In patients treated with infliximab plus MTX, baseline TNC levels significantly correlated with tender joint counts (TJC) at 18 and 54 weeks after initiation of infliximab therapy.

**Conclusions:**

Raised circulating TNC levels are detected in specific inflammatory diseases. Levels are especially high in RA where they may act as a biomarker of bone erosion and a predictor of the effect of infliximab on RA patient joint pain.

## Introduction

Rheumatoid arthritis (RA) is a systemic, progressive autoimmune disease, which affects approximately 1% of the population worldwide. Early treatment within months of the onset of persistent symptoms is recommended and this typically comprises disease-modifying anti-rheumatic drugs (DMARDs) such as methotrexate (MTX). In many patients however, DMARD therapy alone is insufficient to halt disease progression and subsequent treatment with more targeted therapies is now commonplace. In particular, agents that target TNF are now in widespread use. A combination of these biological drugs with MTX can reduce clinical symptoms and disease progression better than either agent alone. However, despite this success, a significant proportion of RA sufferers (approximately 40%) do not respond to this therapeutic approach and these patients would benefit from early prescription of alternative treatments [1].

In combination with a well-defined set of clinical features, a panel of biomarkers is routinely used both in the diagnosis of RA (for example, the presence of rheumatoid factor and/or anti-citrullinated peptide (CCP) antibodies in serum) and the monitoring of disease progression (for example, C-reactive protein (CRP) levels and erythrocyte sedimentation rate (ESR)). While these markers provide valuable information to the clinician, they are poorly predictive of disease prognosis and fail to reliably inform management decisions for individual patients. Consequently, the identification of further easily assayed biomarkers, that are indicative of disease progression or the response of an individual to treatment, would enable the clinician to tailor distinct therapies for each patient [2,3].

Tenascin-C (TNC) is a pro-inflammatory extracellular matrix (ECM) glycoprotein. Its expression in adults is restricted to sites of tissue injury, particularly during phases of inflammation and active tissue remodelling. Expression of TNC is typically a transient event and tissue levels return to normal after the completion of tissue repair. In contrast, persistent expression of TNC is observed in a number of pathologies associated with inflammation and tissue remodelling, including autoimmune diseases such as RA [4,5].

TNC is proposed to act as a damage-associated molecular pattern (DAMP) during RA, where its release upon joint tissue damage induces the synthesis of pro-inflammatory mediators that generate a self-perpetuating cycle of chronic inflammation leading to further joint damage. Injection of TNC directly into the murine synovial joint cavity induces synovial inflammation and in animal models of RA, TNC-deficient mice show rapid resolution of joint inflammation and reduced disease severity when compared to wild type mice [6]. TNC promotes both innate and adaptive immune responses during joint inflammation via a number of different mechanisms. We have previously demonstrated that the C-terminal fibrinogen globe of TNC induces pro-inflammatory cytokine and chemokine production from both primary human macrophages and synovial fibroblasts isolated from RA patients by a mechanism that requires toll-like receptor 4 (TLR4) [6]. TNC has also been shown to mediate cytokine synthesis in murine myeloid cells via activation of α9β1 integrin by a region within the fibronectin type III-like repeats [7]. Moreover, TNC can stimulate pathogenic Th17 cell polarization [8,9] and drives IL-17 synthesis during murine joint inflammation *in vivo *[9].

In RA patients TNC is expressed by the synovial fibroblasts and infiltrating myeloid cells of the diseased joint [10,11], where it is deposited as a dense matrix in the synovial lining layer and where there is also peri-vascular deposition of TNC [11-14]. Furthermore, elevated levels of soluble TNC are found in the synovial fluid of RA patients compared to osteoarthritis (OA) patients and normal healthy individuals [15,16]. To date no reports have looked at TNC in serum from RA patients, and it is unclear whether circulating levels of TNC correlate with disease parameters such as activity, inflammation, bone destruction or response to therapy. To address this question we examined the level of circulating TNC in RA patients from four independent cohorts, as well as patients with other inflammatory diseases that exhibit joint inflammation and arthritis-like symptoms.

## Materials and methods

### Patients

Patients from four separate RA cohorts were used in this study. Cohort A: patient inclusion criteria for this trial have been described previously [17]. Briefly, patients were required to have a diagnosis of erosive RA according to the American college of rheumatology (ACR) 1987 criteria, with symptoms of 0.5 to 3.0 years duration. Twenty-four patients were randomly assigned to one of two groups. One group received infusions of infliximab at 5 mg/kg, at weeks 0 (baseline) 2, 6 and then every 8 weeks through to week 46. The other group received placebo infusions. Both groups received stable doses of MTX. Thereafter, at 54 weeks, those patients in the placebo group received infliximab infusions at weeks 54, 56, 62 and thereafter every 8 weeks. The original infliximab group continued to receive infliximab infusions at the same time points. Blood samples were taken pre-treatment (baseline) and at weeks 6, 18, 52, 58, 70 and 104. Serum was stored at -80°C.

Cohort B: inclusion criteria for entry into the trial were that patients were required to have a diagnosis of RA with disease activity scored at a moderate level or higher. Twenty-seven patients received MTX with either placebo or tranilast (N-(3,4-dimethoxycinnamonyl)-anthranilic acid), an immune-modulatory compound, at either 75 mg/kg or 150 mg/kg *bis in die *(BID). Blood samples were taken at 0 (baseline), 2, 8 and 16 weeks. EDTA plasma samples were stored at -80°C.

Cohort C and cohort D were open cohorts of patients with a diagnosis of RA. Blood samples were collected during visits to outpatient departments and serum was stored at -80°C.

Demographic data were collected from cohorts A and B (Table 1). Patients were recruited anonymously to cohorts C and D and as such no demographic information is available for these groups. The registration details of the controlled trials as are follows: Full title of study: A Phase II, Randomized Multi-Centre, Double-Blind Study of Tranilast with Concomitant Methotrexate (MTX) Compared to MTX Alone in Patients with Active Rheumatoid Arthritis (RA); REC reference number: 09/H0707/26. EudraCT number: 2008-006917-25.

**Table 1:** Demographic data from cohorts A and B

Cohort A		Cohort B				
**Age**		**Age**				

20	38	68	39	64	30	54
60	60	53	57	49	65	66
71	44	53	68	74	66	61
61	49	48	68	64	68	56
46	52	75	51	57	47	80
56	59	73	51	76	52	
39	66	53	58	69	75	
68	51	59	67	34	78	
59	44	60	66	69	66	
47	68	66	74	34	54	
71	45	55	56	71	55	

Age is shown in years. The mean age was 53.36 years (+/- 12.63) for cohort A and 60.24 years (+/-11.63) for cohort B (+/-SD). The median age for cohort A was 54.00 years and for cohort B, 61.00 years. Patients in both cohorts were predominantly female (72.7% in cohort A and 73.5% in cohort B).

Blood samples from patients with systemic lupus erythematosus (SLE), idiopathic inflammatory myositis (IIM), Sjogren's syndrome (SS), psoriatic arthritis and ankylosing spondyloarthritis (AS) were collected during visits to outpatients departments and serum was stored at -80°C. All samples were collected with informed consent in accordance with UK ethical requirements under ethics approval for the collection of research samples from outpatient clinics (cohorts C and D, and disease controls) or under specific ethical approval for clinical trials (cohorts A and B).

### Imaging

In cohorts A and B high-frequency ultrasonography of all 10 metacarpophalangeal joints was performed at weeks 0, 18, 54 and 110 as described previously [17]. Radiographic evaluation of the hands and feet was performed at baseline, 54 and 110 weeks and scored for erosions as described [17].

### Disease activity monitoring

In cohorts A and B, tender joint counts (TJC), swollen joint counts (SJC), CRP, disease activity score (DAS28) and ESR were evaluated at all time points. In cohort D, CRP and anti-CCP were measured at the time of sampling.

### ELISA

Levels of TNC in serum and plasma samples were determined using the human TNC Large (FN III-C) Assay ELISA kit from IBL (Fujioka, Japan). The intra- and inter-assay coefficients of variation for this ELISA are 4.3% and 5.3% respectively, at a value of 3.22 ng/ml. Antibodies to CCP were measured by ELISA (Eurodiagnostica, Malmo, Sweden). Cartilage oligomeric matrix protein (COMP) was measured by ELISA (Biosource, Ghent, Belgium). Type II procollagen (CPII) and marker of type II collagen cleavage (C2C) were measured by ELISA (IBEX, Montreal, Canada). Procollagen III N-terminal propeptide (PIIINP) was measured by ELISA (USCN, Wuhan, China). Hyaluronan was measured by ELISA (R and D systems, Abingdon, UK). All assays were performed according to manufacturers' instructions.

### SDS PAGE and western blotting

1 μl of serum or plasma was electrophoresed on 4 to 12% Bis-Tris pre-cast polyacrylamide gels (Invitrogen, Life Technologies, Paisley, UK). Proteins were transferred onto nitrocellulose membranes (Amersham, GE Healthcare, Chalfont, UK) and visualised using Ponceau S stain (Sigma Aldrich, Gillingham, UK). After washing with TBS/0.01% Tween to remove stain, membranes were blocked in 5% BSA/TBS-Tween for 1 h at room temperature and incubated with primary antibody recognising the N-terminal region of human TNC (MAB 1908, Merck Millipore, Watford, UK) at 1:1000 dilution for 1 h at 37°C. Horseradish peroxidase-conjugated anti-mouse IgG (Dako, Ely, UK) was used as a secondary antibody at a dilution of 1:3000. Bound antibody was detected using the enhanced chemiluminescence kit (Amersham, GE Healthcare) and visualized on Super RX medical x-ray film (Fuji, Bedford, UK).

## Results

### Circulating levels of TNC are raised in RA and other inflammatory conditions

**Figure 1:**
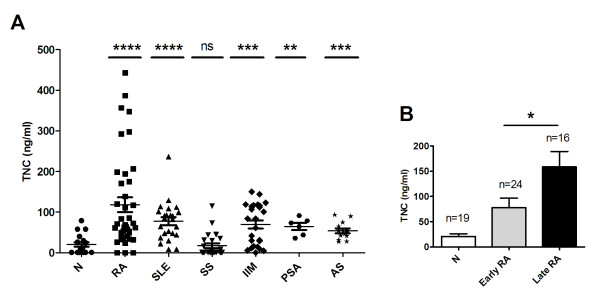
**Circulating tenascin-C (TNC) is raised in rheumatoid arthritis (RA), systemic lupus erythematosus (SLE), idiopathic inflammatory myositis (IIM), psoriatic arthritis (PSA) and ankylosing spondylitis (AS) patients but not in Sjogren's syndrome (SS) and TNC serum concentration increases with disease duration in RA**. (**A**) TNC levels were determined by ELISA in serum samples from healthy controls (N) (*n *= 19), and patients with RA (*n *= 40), SLE (*n *= 24), IMM (*n *= 25), SS (*n *= 24), PSA (*n *= 6) or AS (*n *= 14). Each point represents the mean value of duplicate samples from an individual. Significant differences between patients and healthy controls were analysed by Mann-Whitney two-tailed analysis; ****P < 0.0001, ***P < 0.001, **P < 0.01, *P < 0.05, ns = not significant. (**B**) The RA cohorts were grouped according to disease duration into early (duration < 3 years) and late (duration > 3 years) RA. TNC levels from serum were determined by ELISA. Bars represent the mean plus standard error of the mean (SEM) from pooled data from 19 controls (N), 24 early RA or 16 late RA patients; *P < 0.05 (unpaired *t*-test).

**Figure 2:**
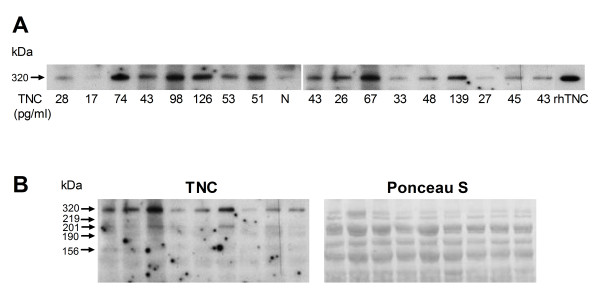
**Western blotting of circulating tenascin-C (TNC) in rheumatoid arthritis (RA) patients**. (**A**) In baseline samples from RA patients one predominant TNC band of 320 kDa was observed (*n *= 18). Corresponding TNC levels detected in the same samples by ELISA are shown in pg/ml under the blot. Low levels of TNC were detected in healthy control plasma (N). rhTNC, 0.05 μg human recombinant TNC. (**B**) In addition to the major band detected at 320 kDa, bands of MW 219, 201, 190 and 156 kDa were present in the plasma of some RA patients upon western blotting with anti-TNC antibodies (TNC). Ponceau S staining of the same membrane shows equivalent protein loading for each RA sample.

The level of circulating TNC in patients with RA, SLE, SS, IIM, psoriatic arthritis, and (AS) was quantified to examine the overall frequency of elevated TNC in rheumatic diseases. Levels of TNC were significantly raised when compared to healthy controls in all conditions examined, with the exception of SS (Figure 1A, Table 2). When TNC serum levels from patients with early disease (cohort A) were compared with those from the other cohorts with predominantly longer disease duration [17], significantly lower levels of TNC were observed in the early stages of disease (Figure 1B), suggesting that circulating levels of TNC increase with duration of disease. Western blotting revealed that the major form of TNC present in RA patient samples has a mass of 320 kDa, (Figure 2A) but minor, smaller, forms of TNC were also observed in some patients (Figure 2B). These bands were not present in normal healthy individuals, nor in blots probed with secondary antibody alone (data not shown).

**Table 2:** Circulating levels of tenascin-C (TNC) are raised in a number of inflammatory conditions

Sample	Mean TNC (ng/ml)	Number of samples	*P*-value (compared to N)	Patients with abnormal TNC, %
Normal healthy subjects (N)	20.7 (95% CI = 33)	19		
Rheumatoid arthritis (RA)	118.2	40	< 0.0001	82.5
Systemic lupus erythmatosus (SLE)	77.8	24	< 0.0001	83.3
Sjogren's syndrome (SS)	16.7	24	0.3489	8.3
Idiopathic inflammatory myositis (IIM)	69.8	25	0.0008	64.0
Psoriatic arthritis (PSA)	64.7	6	0.0026	100.0
Ankylosing spondylitis (AS)	53.8	13	0.0006	76.9

Blood was taken from normal healthy controls (N) and patients with RA, SLE, SS, IIM, PSA and AS, and TNC levels were determined in serum by ELISA. The 95% confidence interval (CI) of TNC levels in normal healthy subjects was determined to be 33 ng/ml. The *P-*value was determined by Student's *t*-test.

### Circulating TNC correlates with joint erosion in RA patients

**Figure 3:**
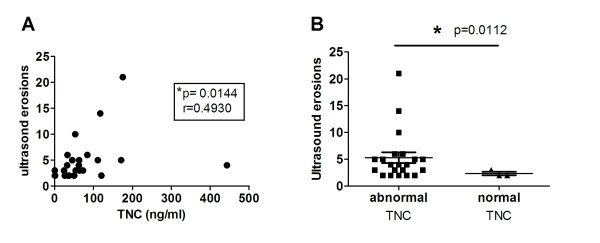
**Levels of circulating tenascin-C (TNC) show a positive correlation with ultrasound-determined erosion scores prior to anti-tumour necrosis factor (TNF) therapy**. (**A**) Levels of TNC in baseline serum samples from early rheumatoid arthritis (RA) patients (*n *= 24) in cohort A were determined by ELISA and compared with erosion scores at baseline as determined by high frequency ultrasonography of 10 MCP joints. Significance was determined using spearman rank correlation analysis. (**B**) Patients with abnormal serum levels of TNC (> 33 ng/ml) (*n *= 21) show higher ultrasound-determined erosion scores at baseline than patients with normal (≤ 33 ng/ml) TNC (*n *= 3); * *P *< 0.05 (*t*-test with Welch's correction).

Given that circulating TNC was raised in RA patients, we examined whether levels of TNC correlated with traditional markers of disease activity and with other potential markers of disease process. Table 3 shows that there was no significant correlation between TNC levels and these biomarkers at baseline sampling. In cohort A both x-ray- and ultrasound-determined erosion data were available. We noted an association between higher baseline TNC levels and higher x-ray-derived erosion scores in baseline samples. Whilst this was not found to be statistically significant (*P *= 0.08), analysis of ultrasound erosion data (considered to be more sensitive for detection of erosions in early RA) from the same dataset revealed a significant correlation between TNC level at baseline and baseline ultrasound erosion scores (*r *= 0.493, *P *= 0.0144) (Figure 3A). In particular, those patients with abnormal levels of TNC at baseline showed significantly higher ultrasound erosion scores than those with baseline normal values at this time point (Figure 3B). These data suggest that circulating levels of TNC do not correlate with currently used clinical markers of disease activity but may be an indicator of erosive joint damage in RA patients.

**Table 3:** Circulating levels of tenascin-C (TNC) do not correlate with any traditional biomarkers of disease in rheumatoid arthritis (RA)

Cohort	Biomarker	Correlation with TNC at baseline?
Cohort A	CRP	No
	Anti-CCP	No
	ESR	No
	Markers of bone and cartilage metabolism: COMP, CPII, C2C, PIIINP, hyluronan	No
	Clinical score: TJC, SJC	No
	Disease activity: DAS28	No
Cohort B	CRP	No
	Clinical score: TJC, SJC	No
	Disease activity: DAS28	No
Cohort C	CRP	No
	Anti-CCP	No
Cohort D	N/A	N/A

TNC levels at baseline (entry into trial) in four separate cohorts of RA patients were analysed by ELISA. CRP, C-reactive protein; anti-CCP, anti-cyclic citrullinated peptide antibody; COMP, cartilage oligomeric matrix protein; CPII, type II pro collagen; C2C, marker of type II collagen cleavage; ESR, erythrocyte sedimentation rate; PIIINP, procollagen III N-terminal propeptide; TJC, tender joint count; SJC, swollen joint count; DAS28, disease activity score 28; N/A, data not available.

### Infliximab treatment transiently reduces serum TNC levels

**Figure 4:**
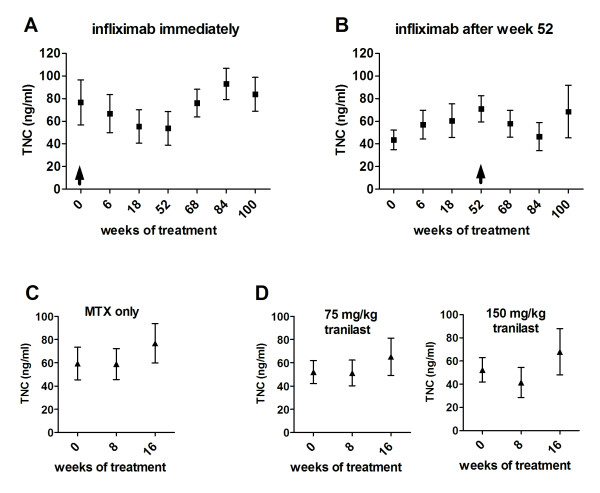
**In rheumatoid arthritis (RA) patients, infliximab therapy results in a temporary reduction in serum tenascin-C (TNC), while treatment with methotrexate (MTX) or tranilast does not**. Blood was taken from RA patients at entry into their trial (week 0) and at various weekly intervals afterwards. Serum (cohort A) or plasma (cohort B) was collected and TNC levels were measured by ELISA. In cohort A patients received infliximab therapy immediately (*n *= 12) (**A**) or after 52 weeks (*n *= 12) (**B**). Arrow shows the start of infliximab therapy. In cohort B, patients received either MTX alone (**C**) or tranilast therapy at 75 mg/kg or 150 mg/kg (**D**) from week 0 (*n *= 9).

Patients in cohort A were divided into two treatment groups, one receiving MTX plus infliximab (5 mg/kg) treatment for 1 year, the other receiving MTX plus placebo for 1 year [17]. After this time both groups received infliximab (5 mg/kg) therapy for a further year. Serum samples from both groups of patients were collected at regular intervals and the effect of treatment on TNC levels was monitored. Patients receiving infliximab + MTX treatment showed a decrease in their levels of circulating TNC (Figure 4A). In contrast, patients receiving only MTX showed a steady increase in the level of circulating TNC between 0 and 54 weeks. It is interesting to note that upon receiving infliximab in the second year of the trial, TNC levels dropped in these patients as well (Figure 4B). In both cases, this infliximab-induced decrease in TNC was transient, with levels rising again approximately 1 year after initiation of infliximab treatment, despite continued therapy (Figure 4A, B). In cohort B, patients treated with MTX alone or with MTX plus tranilast at either 75 mg/kg *BID *or 150 mg/kg *BID *showed a rise in TNC levels similar to that observed in cohort A for MTX alone (Figure 4C, D). These data indicate that the decrease in serum TNC in patients receiving infliximab is due to a specific action of this drug or class of drugs targeting TNF.

### High serum TNC is predictive of unresolved joint tenderness in infliximab-treated patients

**Figure 5:**
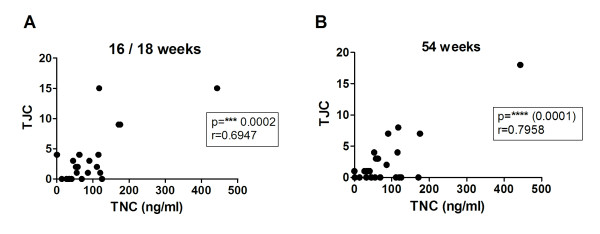
**Tenascin-C (TNC) level at baseline is predictive of future tender joint count in rheumatoid arthritis (RA) patients at both 16 to 18 weeks and 54 weeks after commencing infliximab therapy**. Serum from RA patients in cohort A at baseline (entry into the trial) was analysed for TNC level by ELISA. TNC levels are shown with practitioner-determined tender joint counts in these patients at 16 to 18 weeks (**A**) and 54 weeks (**B) **after receiving infliximab therapy (*n *= 24). Significance was determined by spearman rank correlation analysis.

Despite the major advances provided by therapy with anti-TNF agents such as infliximab, a significant proportion of patients treated with anti-TNF agents do not respond, and continue to acquire joint erosions and increase in disease activity score 28 (DAS28). In the light of the correlation of TNC levels with erosion scores and the effect of infliximab on circulating TNC levels, we asked whether the level of TNC served as a predictor of future disease progression in infliximab-treated patients. There was no relationship between baseline level of TNC or change in TNC level at weeks 18 or 52 after commencement of therapy and response to infliximab. However, a significant correlation was seen between the level of TNC at baseline and the subsequent TJC score in infliximab-treated patients at both 16 to 18 and 54 weeks after the start of treatment (Figure 5). Thus, these data suggest that patients with higher levels of TNC before the initiation of infliximab therapy are more likely to have unresolved joint tenderness despite infliximab therapy.

## Discussion

Over the last two decades the advent of drugs such as anti-TNF used in combination with concomitantly administered MTX has revolutionized the standard of care of many RA patients. However, individual RA patients differ significantly in the clinical manifestation of their disease, as well as in their response to treatment with these drugs. As such, disease diagnosis would ideally be followed by early implementation of the therapeutic regime most likely to be effective in reducing the inflammatory burden in each patient. One major obstacle to this approach is that existing biomarkers of disease cannot predict disease prognosis or patient response to treatment.

Here we examined whether detection of circulating levels of TNC, a pro-inflammatory glycoprotein, may be useful in this respect. TNC is not expressed, or is expressed only at very low levels, in most healthy adult tissues. Little TNC is observed in healthy joints; however, high levels of TNC are detected in the synovia of RA patients [10-14]. Consistent with these data, we found little TNC in the serum of normal healthy subjects, but high levels were detected in the serum of RA patients; more than 80% of patients with RA showed circulating levels of TNC greater than those found in healthy controls. We also observed elevated TNC levels in patients with a range of inflammatory diseases, all of which are associated with chronic arthritis. Thus, raised serum TNC is not specifically associated with RA. Furthermore, raised serum levels of TNC have been reported in diseases with no known arthritic manifestation, including cryptogenic pneumonia, cardiovascular diseases such as myocardial infarction and dilated cardiomyopathy [18,19], chronic hepatitis C [20], inflammatory bowel disease (IBD) [21] and sepsis [22]. Notably, however, we found that patients with SS exhibited no significant increase in serum TNC over healthy controls. Similarly, serum TNC is elevated in some cancers, such as melanoma [23] and ovarian cancer [24], but not in others, such as small lung cell carcinoma [25,26]. These data suggest that high serum TNC is associated with pathological tissue inflammation and remodelling in a wide variety of diseases.

TNC is a large, multimodular molecule. It comprises a number of distinct domains, including an assembly domain, a series of fourteen-and-a-half epidermal growth factor-like repeats, a series of up to seventeen fibronectin type III-like repeats (TNIII), and a fibrinogen-like globe. TNC is encoded by a single gene that is alternatively spliced to create monomers ranging in size from 190 to 320 kDa. This occurs specifically in the TNIII domains; eight of these are constitutively expressed (TNIII1-8) and nine can be alternatively spliced (A1-4, B, AD2, AD1, C and D) [4,27]. There is considerable tissue and disease stage-specific variation in TNC isoform expression. This has been best studied in tumours, where the expression of specific splice variants can predict survival of patients with chondrosarcoma or response to tamoxifen in patients with breast cancer [5]. Here, we found that the predominant variant of TNC in RA serum was 320 kDa in mass, suggesting that this constitutes the large isoform possessing all nine alternatively spliced TNIII repeats. The functional relevance of every alternatively spliced domain is not clear, but their inclusion in TNC variants expressed in RA may contribute to disease pathogenesis. For example, splicing in domains B and D promotes tumour cell proliferation and invasion, and repeats A to D have been shown to confer tumour cell survival (reviewed in [5]). In addition to the major band at 320 kDa, we also observed minor bands of a lower mass in some patients. These data could suggest that a small amount of differently spliced TNC isoforms can be expressed in RA, that a minor proportion of circulating TNC is subject to proteolytic degradation or that a minor proportion of circulating TNC derives from proteolytic cleavage of the intact ECM of the RA synovium. Further studies are needed to identify more precisely the post-transcriptional modification of TNC occurring in the joints in RA.

Localized TNC expression has previously been shown to associate with disease activity; elevated levels of TNC in the tumour stroma correlate with metastasis and poor prognosis in both breast and brain cancer [27,28]. In the heart, both cardiac tissue levels of TNC in the extracellular matrix and circulating TNC correlate with disease severity in acute myocardial infarction, dilated cardiomyopathy and myocarditis [27]. Furthermore, high levels of circulating TNC in chronic hepatitis C adversely correlate with disease outcome [20], and serum TNC levels in patients with ulcerative colitis (UC) correlate with disease activity and reflect improved disease symptoms after treatment of both UC and IBD [21]. When we examined a link between raised serum TNC and RA disease activity, we found that TNC levels specifically correlated with joint erosion, but not with any other disease parameter. Thus, circulating TNC is not associated with clinical markers of inflammation or with DAS28 but may be an indicator of erosive joint damage in RA patients.

TNC expression is specifically associated with sites of tissue injury where it is induced in response to a range of stimuli, including mechanical stress, pro-inflammatory cytokines, growth factors, hypoxia and reactive oxygen species [4]. High levels of circulating TNC in patients with erosive RA therefore may appear as a consequence of advanced joint tissue destruction. The expression of TNC itself however provokes further tissue injury, potentially via a number of different mechanisms, suggesting that raised serum TNC in RA may serve both as cause and effect of active joint erosion. In murine models of joint disease TNC drives the synthesis of pro-inflammatory cytokines and chemokines such as TNF, IL6 and IL8 [9], which in turn mediate the synthesis of tissue-destructive enzymes. TNC can also directly promote collagenase expression in rabbit synovial fibroblasts [29]. Moreover, TNC is required for the induction of IL-17 in inflamed murine joints [9], a cytokine that directly mediates joint tissue degradation by activating bone resorbing osteoclasts, inhibiting cartilage matrix synthesis and inducing proteases [30]. IL-17 is also found at high levels in RA patients with advanced levels of joint damage [30]; it has also been shown to drive autonomous, TNF-independent erosive disease in mice [31,32] and TNF therapy is anecdotally reported to prevent erosion less effectively in RA patients with high IL-17 levels [30]. As such, patients with high serum TNC may benefit from therapeutic blockade of IL-17 activity rather than inhibition of TNF.

Our data reveal that TNC levels increase over the course of RA progression. We also observed that infliximab treatment of RA produces a rapid reduction in serum TNC levels, while patients receiving MTX alone have a progressive increase in circulating TNC. TNF induces TNC expression in chondrocytes and fibroblasts [33,34] and so our data may reflect the fact that inactivation of TNF removes a stimulator of TNC expression. This would explain the observation that treatment with tranilast is unable to modulate TNC expression in the same way as infliximab. However, the effect of infliximab on TNC levels is temporary causing only a transient drop in TNC, which returns to abnormal levels after 1 year. This rebound in circulating TNC despite continued treatment, suggests that alternative mechanisms of TNC induction can dominate during TNF suppression. It may be interesting to examine the effect of other biological drugs on TNC levels in RA patients. In particular, as levels of TNC correlate with those of IL-6 in the RA synovium [10], and as IL-1 has been shown to induce TNC expression in cultured synovial fibroblasts [11], tocilizumab and anakinra may have similar effects on TNC expression.

## Conclusions

Despite the improved outcome for many RA patients treated with anti-TNF agents such as infliximab, approximately 30 to 50% of patients do not benefit, and their disease continues to progress. We found that in patients treated with infliximab and MTX, high serum TNC was predictive of a poor response of joint tenderness to infliximab. This finding that the level of TNC in serum before beginning infliximab treatment acts as a predictor of tender joint count as far as one year in advance, may allow us to begin to distinguish between patients who are likely to benefit from anti-TNF treatment.

## Abbreviations

ACR: American College of Rheumatology; AS: ankylosing spondylitis; CRP: C-reactive protein; anti-CCP: anti-cyclic citrullinated peptide antibody; BID: *bis in die *(twice daily); COMP: cartilage oligomeric matrix protein; CPII: type II pro collagen; C2C: marker of type II collagen cleavage; DAMP: damage-associated molecular pattern; DAS28: disease activity score 28; DMARD: disease-modifying anti-rheumatic drug; ECM: extracellular matrix; ELISA: enzyme-linked immunosorbent assay; ESR: erythrocyte sedimentation rate; IBD: irritable bowel disease; IIM: idiopathic inflammatory myositis; IL: interleukin; MTX: methotrexate; OA: osteoarthritis; PIIINP: procollagen III N-terminal propeptide; PSA: psoriatic arthritis; RA: rheumatoid arthritis; SLE: systemic lupus erythematosus; SS: Sjogren's syndrome; SJC: swollen joint count; TJC: tender joint count; TLR: toll-like receptor; TNC: tenascin-C; TNF: tumour necrosis factor; TNIII: fibronectin type III-like repeats; UC: ulcerative colitis.

## Competing interests

The authors declare that they have no competing interests.

## Authors' contributions

THP performed experiments, analysed data and drafted the manuscript. PJC contributed to study design, data analysis and manuscript review. AMP and VN performed experiments and helped with manuscript review. PCT conducted clinical trials, and contributed to study design, data analysis and drafting the manuscript. KSM conceived the study and contributed to study design, data analysis and drafting the manuscript. All authors read and approved the final manuscript.
